# Lisfranc Sports Injuries: What Do We Know So Far?

**DOI:** 10.7759/cureus.48713

**Published:** 2023-11-13

**Authors:** Godsfavour C Maduka, Divinegrace C Maduka, Naeem Yusuf

**Affiliations:** 1 Trauma and Orthopaedics, Lister Hospital, East and North Herts National Health Service (NHS) Trust, Stevenage, GBR; 2 Major Trauma, Queens Medical Centre, Nottingham University Hospitals National Health Service (NHS) Trust, Nottingham, GBR; 3 Plastic Surgery, Lister Hospital, East and North Herts National Health Service (NHS) Trust, Stevenage, GBR

**Keywords:** lisfranc sport injury, lisfranc injury, primary arthrodesis, lisfranc injury classifications, return-to-play, neglected foot injury, surgical fixation, lisfranc fracture

## Abstract

Lisfranc sports injuries include tarsometatarsal joint injuries, which may be accompanied by fractures. They most commonly occur due to a blow or axial force.* *The aim of this review is to assess the current standards for surgical intervention in Lisfranc injuries resulting from sports-related accidents.

This evaluation will cover the timing of treatment, the recovery process, and the appropriate timing for a return to normal sporting activities. This research was done via an analytical review of current literature. Methods included a structured search strategy on PubMed, Science Direct, and Google Scholar. The collated literature was processed using formal inclusion or exclusion, data extraction, and validity assessment.

Joint involvement and severity were taken into account while classifying Lisfranc injuries. The primary fixation and fusion techniques for Lisfranc injuries were compared, and the surgical management of these injuries was examined in all of the literature. Treatment recovery times were examined, and the results were talked about. A variety of injuries, from minor sprains to serious fractures and rips, make up Lisfranc injuries. Although open reduction internal fixation (ORIF) in combination with primary arthrodesis (PA) is now thought to be the optimum course of treatment, its acceptance has increased.

Patients with Lisfranc injuries can usually expect excellent outcomes and the return of joint function to its pre-injury form if the injury is appropriately assessed and treated. Lisfranc injuries are manageable and have a good recovery time if not neglected. The outcomes of management and surgical options are also quite satisfactory.

## Introduction and background

Lisfranc sports injuries, as described in the literature, typically entail a partial or complete disruption of the tarsometatarsal joint's articular congruency, occasionally accompanied by fractures [1]. Lisfranc sports injuries range from high-energy trauma to common ligament injuries in sports [2]. The term "Lisfranc injury" is named after a French doctor in 1815 who was the first to describe this type of injury and detail a surgical amputation at that level [3]. Lisfranc injuries are rare, accounting for 0.2% with a 20% diagnostic delay. Early identification and treatment are crucial for positive outcomes and preventing long-term complications and functional limitations [4].

Lisfranc injuries in sport often result from an indirect mechanism involving axial longitudinal force on the plantar-flexed and slightly rotated foot, followed by forceful abduction or sudden twisting during athletic activity [5]. The Lisfranc joint's inherent stability, with solid bones and strong ligaments, limits its mobility [6]. An increased occurrence of dorsal dislocations in Lisfranc injuries has been seen, and this phenomenon is attributed to the greater thickness of the plantar ligament complex [7]. Almost 20% of the complicated tarsometatarsal joint injuries are misdiagnosed on first examination, especially when caused by low-energy trauma [8].

Diagnosis of Lisfranc sport injuries is achieved using X-rays, CT scans, or MRIs depending on injury and pain [9,10] and can be facilitated by bone scintigraphy [11]. Classifying Lisfranc injuries is vital but challenging due to their wide range and variability [7]. Various classification systems have been devised, ranging from Quenu and Kuss in 1909 [12], while a classification based on the medial, middle, and lateral columns was introduced by Chiodo and Myerson in 2001 to address the shortcomings of Hardcastle and Myerson's 1986 [13,14] classification, which attempted and largely failed to dictate treatment and prognosis.

Lisfranc injury treatment options include non-operative (cast immobilisation) or surgery. The choice depends on the injury's nature and severity. Surgery includes joint-saving procedures like open reduction internal fixation (ORIF) with pins, plates, screws, or K-wires, aiming to preserve joint integrity [15]. Surgical options may include midfoot primary arthrodesis (PA) and fusion, aiming to stabilise the joint by fusing it to restrict movement [16]. These injuries are rare but increasing in athletes, highlighting the need to optimise treatment strategies for this patient group [17]. The aim of this review is to assess the current standards for surgical intervention in Lisfranc injuries resulting from sports-related accidents. This evaluation will cover the timing of treatment, the effects of neglected injuries, the recovery process, and the appropriate timing for a return to normal sporting activities.

## Review

Methodology and materials

An analytical literature review is used in this research paper with key words such as Lisfranc fracture, Lisfranc sport injuries, and neglected foot injuries. It is used to gather data from a variety of sources, including online databases, orthopaedic journals, and articles primarily from PubMed, Google Scholar, Web of Science, NCBI, and UpToDate. These sources assess and discuss the subject, allowing for an evaluation and the subsequent formulation of a well-supported conclusion about Lisfranc sports injuries and their various treatment options and outcomes. It produced an analytical literature evaluation in accordance with acknowledged worldwide standards and methodological norms.

Inclusion and Exclusion Criteria

**Figure 1:**
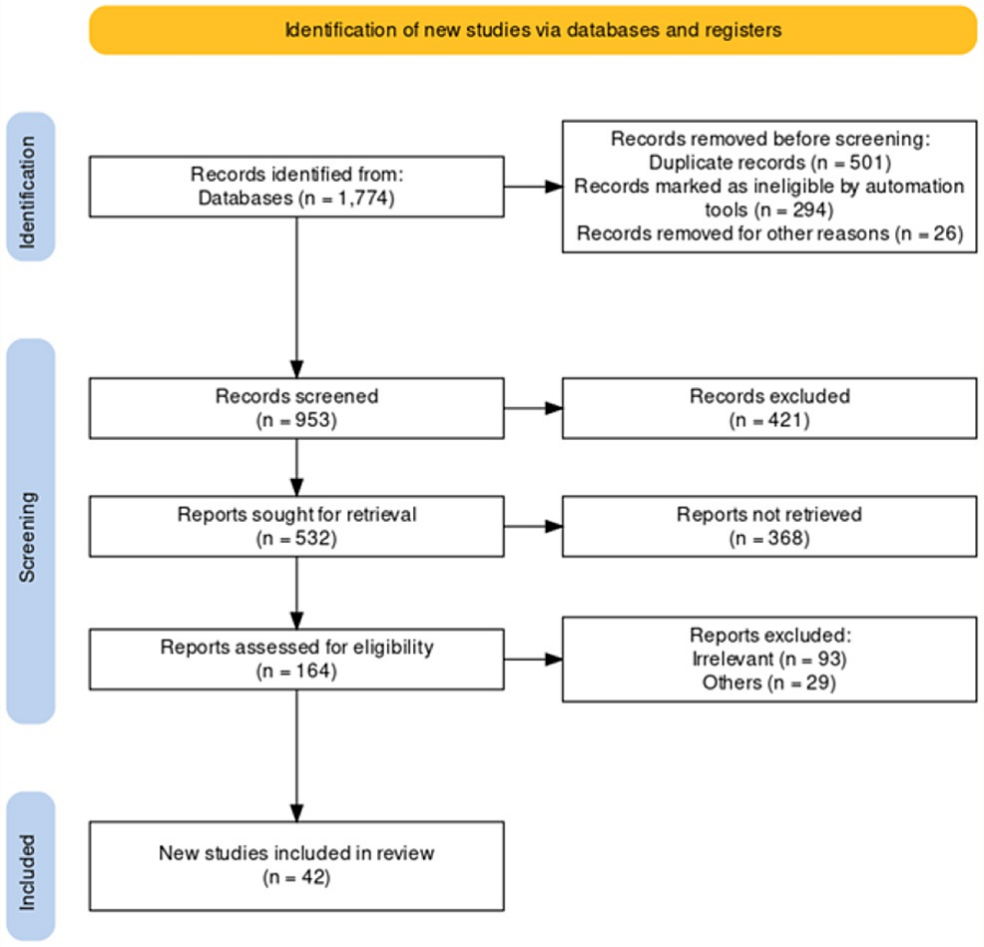
PRISMA flow diagram of the included studies

The inclusion criteria used in this literature review are: (i) only studies that looked at patients between the ages of 15 and 65 who had no other substantial chronic comorbidities or congenital abnormalities. (ii) We did not consider the research participants' race, gender, or ethnicity to be important criteria for including or excluding a study from our analysis. (iii) All randomized controlled clinical trials assessing and contrasting any of the main Lisfranc treatments that have been given approval in the literature. Studies assessing all Lisfranc injuries were taken into consideration for inclusion if they satisfied all the other criteria for inclusion. The exclusion criteria for the study say (i) Studies with contradictory, missing, or insufficient data were disregarded. (ii) All studies that were not published, regardless of the type of study, were excluded. The linked bibliography lists all the papers and articles that were cited. A total of 42 studies were included in the review. The PRISMA flow chart of the included studies is given below (Figure 1).

Data Collection and Organisation

During the screening, papers were evaluated based on inclusion and exclusion criteria. Studies failing to meet inclusion or exclusion criteria were omitted. Irrelevant or inaccessible information was also excluded. Qualifying studies with similarities were grouped with no consideration of race or gender. Instead, studies addressing similar injuries or using comparable classification methods were grouped for efficient data analysis and result generation.

Data Analysis

After gathering and organising the data from clinical studies, a comparative analysis was performed. Studies with similar experimental protocols and controlled variables, including patient age, gender, and activity level, were examined. The results were compared to identify the most effective treatment within each group. Furthermore, studies exploring treatment methods for similar injuries were compared, particularly for injuries of similar severity, when using consistent classification systems. An assessment of the quality and risk of bias in the included randomized controlled trials (RCTs) based on the Cochrane Risk of Bias Tool was used as part of the methodology. To assess the quality of the included observational studies, the Newcastle-Ottawa Scale was used. Each outcome’s evidence quality was determined via the application of the Grading of Recommendations Assessment, Development, and Evaluation (GRADE) approach.

Review

Classifications of Lisfranc Injuries

In the literature, 14 classifications for tarsometatarsal injuries have been discussed [18]. Different classification systems describe Lisfranc injuries based on TMT joint congruence and MT base displacement direction. Quénu and Küss introduced an enduring classification in 1909 [19]. The classification prioritised metatarsal displacement direction, dividing injuries into homolateral, isolated, or divergent categories. It splits the foot into the first metatarsal (medial column) and the lesser lateral metatarsals (commonly called the "spatula"), which could dislocate separately or together. The modified version of this classification was presented by Hardcastle et al. in 1982 [20] and by Myerson et al. in 1986 [21]. The Lisfranc injury classification includes Type A, Type B1, and Type B2, delineating incongruence levels in the tarsometatarsal joint complex. Type A signifies complete incongruence, while Types B1 and B2 involve partial incongruence with medial or lateral dislocation of specific metatarsals. Types C1 and C2 depict divergent injuries with partial or total displacement. Despite these classifications categorizing Lisfranc injuries based on their specific features, they offer limited guidance in treatment and prognosis planning. Studies show a minimal correlation between the Myerson et al. [21] classification system and treatment selection. Practitioners often consider factors like stability, displacement, and isolated ligament involvement when choosing surgical treatments rather than relying on a specific classification system [22]. Myerson himself has acknowledged the complexity of existing classifications and their limited contribution to treatment decision-making [14]. This suggests that other factors and considerations hold more weight in determining the appropriate approach for Lisfranc injuries.

In 2001, Chiodo and Meyerson classified tarsometatarsal joint injuries based on a three-column theory [14]. This classification divides the foot into three columns: the Lateral Column, the Medial Column, and the Middle Column. It focuses on mid-foot motion segments, treating metatarsals within each column as a functional unit. Anatomical variations in rigidity, motion range, and tolerance of post-treatment incongruence within these columns carry important prognostic implications, underscoring the classification's significance in managing tarsometatarsal injuries [14].

Prior to Nunley and Vertullo's 2002 paper, there was no established classification system for low-velocity Lisfranc injuries, especially those without diastasis, common in midfoot sprains. Their three-stage classification introduced in the paper takes into account clinical examination, radiography, and bone scintigraphy. Stage I signifies clinical mid-foot pain with normal radiographs and positive scintigraphy. Stage II involves clinical pain with a 1-5 mm diastasis in radiographs but no loss of the plantar arch, while Stage III denotes a diastasis over 5 mm in radiographs with a distinct loss of plantar arch height. Stage I suggests six weeks of immobilization in a non-weight-bearing cast, whereas Stages II and III often call for ORIF surgery, followed by a non-weight-bearing time [11].

In 2016, a simplified Lisfranc injury classification was introduced that categorizes injuries into subtle and evident types. Subtle injuries encompass Type I (Lisfranc ligament sprains with a <2 mm distance between the first and second metatarsal bases) and Type II (complete ligament rupture or ≥2 mm diastasis). This uncomplicated classification aids in prognosis and guides treatment decisions, whether they lean toward conservative or surgical approaches. [23] This classification seems to be the most practical and clinically important classification currently described in the literature, as it directly guides management and intervention. 

Surgical Management of Lisfranc Sport Injuries

Standardising primary management has been difficult given the heterogeneity observed in the severity of sports-related Lisfranc injuries [24]. Surgical intervention is recommended for more severe Lisfranc injuries, such as Nunley and Vertullo Stage 2/3 injuries [11] and Romero classification subtle type 2 and evident injuries [23]. The objective of the surgery is to achieve anatomical reduction, which helps restore articular function. This approach is crucial for ensuring the best possible outcome in these complex Lisfranc injury cases [25]. Specific surgical interventions and techniques can be guided to some extent by secondary objectives, such as the preservation of articular cartilage to prevent post-operative osteoarthritis [26].

However, the ultimate determination of specific surgical techniques is primarily based on their ability to achieve the primary objective of achieving adequate anatomical reduction [27]. Surgical approaches are often tailored to individual patients and the severity of their injuries [28]. Current literature highlights the heterogeneity of surgical options available, emphasizing the need for a personalized approach to treatment based on the unique characteristics of each Lisfranc sport injury case.

Some authors recommend attaining anatomic reduction via closed reduction before fixation [29,30], but common practice tends towards open reduction as it enables the extraction of tissue and a clear view of the injury [31]. Although a dorsal approach may be required for more serious injuries, open reduction utilizing a longitudinal technique is often the primary surgical treatment for Lisfranc joint problems [32]. Internal fixation is carried out using Lisfranc (trans-articular) screws placed in the medial and middle columns, securing them to the corresponding metatarsal bones [33]. For injuries to TMT (tarsometatarsal) IV or V joints, the preferred surgical intervention, as per the reviewed literature, is temporary fixation with Kirschner wires (K-wires) following open reduction [34]. These surgical techniques aim to achieve anatomical reduction and stability in Lisfranc injuries.

As a result of the reported 40%-90% incidences of posttraumatic osteoarthritis after fixation by the standard method [35]. The use of an extra-articular dorsal plate for TMTJ fixation is more recently described in the literature. According to published research, using this alternative procedure instead of fixing the TMTJ with intra-articular screws to protect the articular cartilage lowers the risk of developing arthritis [36]. By using this technique, the TMTJ is stabilized in the anatomic position while the articular cartilage is preserved, and a rigid fixation is provided [37]. Irritation of the soft tissues is noticed. Sometimes, a second surgery is required [38]. Trans-articular screws can still be used in patients who have a high expected likelihood of post-operative arthritis, such as in cases where preservation of articular cartilage is impossible or futile [39].

Primary fusion is typically reserved for cases involving comminuted intra-articular fractures, where the risk of posttraumatic arthritis is high [2]. In situations where it is not possible to achieve adequate reduction and stabilization of the comminuted articular surfaces, primary partial arthrodesis procedures may be indicated. This can involve fusing the medial column, prioritizing the need for proper anatomical reduction over the preservation of articular cartilage [40]. Secondary arthrodesis may also be considered for patients who require a transition from fixation to fusion due to poor outcomes and ongoing osteoarthritis, with the goal of improving functionality in the affected joint [41]. These decisions are made based on the specific characteristics of the Lisfranc sports injury and the patient's individual needs.

Fixation vs. Primary Fusion Outcomes in Lisfranc Injuries

The two main surgical management methods for Lisfranc injuries described in the literature are Open Reduction and Internal Fixation (ORIF) and Joint Arthrodesis [42]. Recent literature supports primary arthrodesis as a viable treatment, potentially reducing pain and improving joint stability for superior long-term outcomes compared to ORIF [43]. Studies comparing acute Lisfranc lesions treated with primary arthrodesis or ORIF consider factors like hardware removal, patient-related outcome measures (e.g., VAS and AOFAS scores), anatomic reduction rates, and return to functionality. These studies inform clinical decisions regarding the effectiveness and safety of primary arthrodesis versus ORIF for Lisfranc joint fractures [42].

Ly and Coetzee's 2006 study was among the earliest to directly compare primary arthrodesis with open reduction and internal fixation (ORIF). Over an average 42.5-month follow-up period with 41 patients, they found an anatomic reduction in 18 of 20 patients in the ORIF group and 20 of 21 in the arthrodesis group. [44] A Smith N., Stone C., Furey study in 2016 [45] concluded that both ORIF and fusion had the same outcomes, and the ratio was found to be 1.48 [45].

Cochran et al. (2017) conducted a study on PA vs. ORIF in athletes with Lisfranc injuries. He concluded that PA had less recovery time as compared to ORIF [46].

Multiple studies compared ORIF and fusion for Lisfranc injuries. Smith et al. (2016) found no clear preference based on patient-reported outcomes, with a standard mean difference of 0.50, indicating similar results between the two approaches [45].

Magill et al. (2019) identified a notable disparity in persistent pain scores but no significant differences in VAS pain scores, AOFAS scores, or infection rates between the ORIF and primary arthrodesis (PA) groups [47].

Han et al. found that in their study, AOFAS ankle-hindfoot scores were lower in the ORIF group compared to the arthrodesis group. Furthermore, the postoperative VAS score was significantly lower in the arthrodesis group compared to the ORIF group [43].

Almost all the literature analysed agrees that ORIF is associated with statistically significant higher rates of revision and hardware removal seen between the two methods [48]. Ly and Coetzee [44] reported that 16 out of 20 ORIF patients and 4 out of 21 PA patients had to remove implants. Follow-up X-rays showed worsening alignment, deformity, and joint degeneration in 15 of the 20 ORIF patients, leading to seven requiring arthrodesis conversion [34].

The availability of high-quality evidence is limited, and more RCT findings are required [49]. The reviewed literature indicates that primary arthrodesis (PA) may reduce secondary surgeries implant removal and hasten return to activity compared to ORIF. However, the impact of hardware removal on additional procedures is noteworthy. PA also shows potential for improved functional outcomes. Yet, these findings are somewhat contentious due to variations in study quality, non-randomized data pooling, diverse patients, surgical and post-op care differences, and various injury mechanisms and sports, contributing to study heterogeneity [50].

Timing of Lisfranc Sports Injuries Treatment

The optimal timing of surgery for Lisfranc injuries remains a topic of debate in the medical literature. While several studies have suggested that earlier treatment intervention, [51] ideally within six weeks of injury, tends to yield better outcomes and prognosis [7], there are also conflicting studies that report no significant statistical difference between acute (early) and delayed (beyond six weeks) surgical intervention [52].

Moracia-Ochagavía I, Rodríguez-Merchán EC (2019) [4] reported that discharge must be after 10 to 15 days with the wrinkling of skin [4]. Myerson and Cerrato study (2008) [53] supports Hardcastle et al.’s (1982) findings [20]. They are both proponents of surgical intervention within six weeks due to post-treatment patient-reported outcomes. According to Frankle et al.’s (2003), ORIF performed within six weeks following injury has produced positive outcomes [54].

Hardcastle et al. study [21] determined that patients treated after six weeks had worse outcomes than those treated within six weeks [20]. Myerseon and Cerrato (2008) found that the longer the wait to treat the initial injury, the greater the difficulty of obtaining a satisfactory anatomic reduction of Lisfranc injuries [22]. These findings, along with the finding that soft tissue scaring at eight weeks tends to make reduction without tissue resection impossible, resulted in the study supporting intervention within six weeks.

Contrary to this, Kuo et al. [22] could not distinguish between patients who received an acute vs. delayed diagnosis and therapy in terms of outcome scores. Feng et al. [55], who treated Lisfranc injuries a mean of 4.8 months after the first injury, corroborated these findings. The 13 of the 15 patients where they maintained reduction had AOFAS scores that were comparable to those of the acutely treated patients in Kuo's study cohort [22].

Outcome and Management of Neglected Lisfranc Injuries

Lisfranc sports injuries frequently result in painful malunion, osteoarthritis, broad-spectrum deformity/disability, chronic instability, and excessive socioeconomic costs associated with repeat surgery if they go untreated or the primary treatment fails due to flawed anatomical reduction, implant failure, or ineffective conservative measures [52]. Surgical interventions for neglected Lisfranc injuries are often associated with poor outcomes [55], resulting from a great deal of soft tissue dissection, articular degeneration brought on by joint malalignment or deformity, and inadequate stability due to soft tissue contraction and rounding of ligament edges [52].

Joint arthrodesis and staged reconstruction are the two main methods suggested to provide the best management outcomes [56]. Staged reconstruction employing spanning external fixators to gradually distract soft tissues and tarsal bones is more appropriate for treating elderly or untreated Lisfranc injuries than single-stage surgical release, according to research by Pin Feng, Ya-Xing Li, and colleagues in 2017 [55,48]. Although this method has a longer treatment cycle and is more expensive, it improves safety and effectiveness by avoiding the many risks and consequences.

Neglected Lisfranc sports injuries often result in advanced arthritis fixed deformity or neuropathic deformity such as intractable pain. Joint arthrodesis has been shown to improve functional scores such as AOFAS in these patients by Komenda et al. [57]; it has been recommended by Pin Feng, Ya-xing Li, and colleagues [48] in patients with severe articular injuries or solely ligamentous injuries as a preferable therapeutic choice to reduce the likelihood of repeated subluxation and arthritic progression [1].

Return to Play After Lisfranc Sports Injuries

The literature strongly supports the idea that prompt and appropriate treatment of Lisfranc sports injuries causes most patients to return to their athletic activities [58]. Studies have consistently shown that individuals who receive proper treatment for these injuries can typically resume their activities within a mean timeframe of 28-35 weeks [58]. Moreover, they often achieve a level of performance comparable to their pre-injury status.

However, it's crucial to acknowledge that Lisfranc sports injuries, while generally manageable, can still have adverse outcomes in some cases. Despite adequate management, a subset of individuals may experience decreased post-injury performance, be forced to retire from their sport, or struggle to continue their pre-injury level of activity. These difficulties underscore the complexity of Lisfranc sport injuries and the value of tailored treatment and recovery to improve athletic outcomes [59].

Research findings indicate that most athletes can successfully return to normal play after Lisfranc sports injuries, with a typical recovery timeline of 18-24 weeks for training and 21-31 weeks for competitive play. Athletes with ligamentous injuries tend to return nearly four weeks earlier than those with bony injuries [4].

MacMahon et al. (2016) examined 38 young patients (average age 31.8 years) who underwent primary partial arthrodesis for Lisfranc injuries, assessing their return to physical activities. However, the study also identified post-operative considerations, including reduced functionality. Approximately 25% reported reduced activity participation, and 34% faced increased activity difficulty [24].

In a study by Bryan Vopat et al. (2019), one surgeon repaired 11 athletes who suffered from Lisfranc injuries with ORIF and screw fixation. Eighty percent of the 10 patients followed continued their sports activities, taking almost 29.4 weeks, ranging from 22 to 52 weeks to return. The athletes returned to approximately 87% of their previous function. However, unlike many other studies, most of these patients still reported some residual pain upon returning to their sports [58].

A 2021 meta-analysis by Bolk et al., which included 15 trials on primary partial arthrodesis, surgical fixation, and conservative treatment for Lisfranc injuries, provided encouraging outcomes for sports-related outcomes. Notably, 93% to 94% of athletes could return to some level of sports activity, and a significant proportion (ranging from 74% to 88%) were able to resume their pre-injury level of sports participation. These findings offer valuable insights for patients, aiding in their understanding of expected outcomes with different Lisfranc injury treatments [24].

## Conclusions

Lisfranc sports injuries are composed of a range of injuries, from mild sprains to severe fractures and tears. While primary arthrodesis in conjunction with ORIF is currently the best course of treatment, it has grown in popularity. This is probably because it is able to achieve and maintain adequate anatomical reduction while avoiding ORIF's disad vantages of healing slowly and the need for secondary surgery for implant removal. As long as the injury is correctly evaluated and treated in a timely fashion, patients with Lisfranc injuries can typically anticipate positive outcomes and the use of joints in the previous pre-injury manner.
